# Collaboration between physicians and a hospital-based palliative care team in a general acute-care hospital in Japan

**DOI:** 10.1186/1472-684X-9-13

**Published:** 2010-06-15

**Authors:** Nanako Tamiya, Mikako Okuno, Masayo Kashiwakgi, Mariko Nishikitani, Etsuko Aruga

**Affiliations:** 1 Department of Health Services Research, Graduate School of Comprehensive Human Care Sciences, University of Tsukuba, Tenno-dai, Ibaraki 305-8575 Japan; 2 Department of Hygiene and Public Health, Teikyo University School of Medicine, 2-11-1 Kaga, Itabashi-ku Tokyo 174-8605 Japan; 3 Department of Palliative Care, International Medical Center of Japan, Toyama, Shinjuku-ku Tokyo 162-8655 Japan

## Abstract

**Background:**

Continual collaboration between physicians and hospital-based palliative care teams represents a very important contributor to focusing on patients' symptoms and maintaining their quality of life during all stages of their illness. However, the traditionally late introduction of palliative care has caused misconceptions about hospital-based palliative care teams (PCTs) among patients and general physicians in Japan. The objective of this study is to identify the factors related to physicians' attitudes toward continual collaboration with hospital-based PCTs.

**Methods:**

This cross-sectional anonymous questionnaire-based survey was conducted to clarify physicians' attitudes toward continual collaboration with PCTs and to describe the factors that contribute to such attitudes. We surveyed 339 full-time physicians, including interns, employed in a general acute-care hospital in an urban area in Japan; the response rate was 53% (*N *= 155). We assessed the basic characteristics, experience, knowledge, and education of respondents. Multiple logistic regression analysis was used to determine the main factors affecting the physicians' attitudes toward PCTs.

**Results:**

We found that the physicians who were aware of the World Health Organization (WHO) analgesic ladder were 6.7 times (OR = 6.7, 95% CI = 1.98-25.79) more likely to want to treat and care for their patients in collaboration with the hospital-based PCTs than were those physicians without such awareness.

**Conclusion:**

Basic knowledge of palliative care is important in promoting physicians' positive attitudes toward collaboration with hospital-based PCTs.

## Background

Among the more than 1 million Japanese who die from various causes every year, over 320,000 (about 32%) succumb to cancer, which has been the leading cause of death since 1981[1]. The number of cancer deaths has been increasing and is expected to reach about 470,000 in 2020[2]. In the context of the current situation in Japan, urgent action is necessary to provide comprehensive treatment for cancer and appropriate palliative care.

The concept of palliative care in Japan, however, lags far behind that in other industrialized countries. In 1984, our government adopted strong measures to improve the diagnosis and treatment of cancer, but no plan for palliative care was established. The belated introduction of palliative care by the government has caused numerous misunderstandings about and barriers to palliative care in Japan. For example, a population-based survey found that bereaved families in Japan who lost a family member to cancer believed that "opioids shorten life" (38%)" and that "opioids cause addiction" (31%)[3]. In 2005, another survey examined 630 bereaved family members of cancer patients who had used palliative care units (PCUs) in Japan and found that belated referrals to specialized palliative care services were caused by "pessimistic images about palliative care" among family members, such as "it means someone is fated to die" and "it is a final step when there is no other way to cure patients."[4]

Lack of familiarity with palliative care affects both the general population and physicians. In 2003, a national survey asked 3,147 Japanese physicians, including those working in all the PCUs and those randomly selected from all hospitals and clinics, about the World Health Organization analgesic ladder (WHO Ladder)[5]. One-quarter of physicians responded that they did not know about the ladder, and 43% of physicians responded that they knew or knew quite a lot about the ladder. Currently, Japanese medical specialists undergo no systematic training about the introduction of palliative care. Indeed, only a few physicians have received formal education on palliative care, and most of these were trained abroad or self-educated via academic papers and conferences. It has been reported that more than half of all patients and bereaved families felt that the timing of their referrals to palliative care units was late or too late[6]. In the context of this background, the Japanese Society for Palliative Medicine is preparing for the accreditation of palliative care in the near future. In addition, the Ministry of Health, Labour and Welfare indicated in 2007 that all physicians who treat cancer must become educated in basic palliative care within the next 10 years[7].

Two main types of specialized palliative care services are offered in Japan. One is the palliative care unit (PCU); 178 certified PCUs (3,417 beds) were listed in 2007[8]. Care in a PCU has been covered by the Japanese health insurance system since 1990, and the per-person reimbursement to date has been 37,880 yen (US$316)/day. The number of certified PCUs is increasing, but many difficulties interfere with establishing PCUs, including a lack of full-time physicians, insufficient nursing staff, and inadequate facilities.

The other type of specialized palliative care service is the hospital-based palliative care team (PCT). PCTs have been developed and evaluated in many Western countries[9-14]. In Japan, a certified PCT must meet certain conditions with respect to the facilities and staff: the hospitals must be accredited by the Japan Council for Quality Health Care, a private sector not-for- profit organization resembling the Joint Commission in the United States, and the team must include a palliative care physician, a psychiatrist, a pharmacist, and a specialized palliative care nurse[15]. Such team services have been offered since 2002, reflecting the WHO announcement that palliative care should be provided early in the course of an illness rather than only at the end of life[16]. The Japanese health insurance system has reimbursed 2,500 yen (US$21)/day/person to certified PCTs since that time.

Unlike PCUs, PCTs can provide care in acute-care hospitals early in the course of an illness in conjunction with other therapies that are intended to prolong life, such as chemotherapy or radiation therapy. In 2007, five years after the WHO modified the definition of palliative care, the Cancer Control Act[17] was enacted in Japan. This act stipulated that palliative care be available early in the course of an illness to improve patients' quality of life (QOL). At about the same time, the government published the Guide for the Improvement of Regional Cancer Centres (*Gan renkei shinryou kyoten byouin nikansuru shishin*)[18] to promote the functioning of regional cancer centres (*Chiiki gan sinnryou renkei kyoten byouin*) in collaborative medical care. According to these guidelines, each of these cancer-specific hospitals must establish a PCT. As a result of the Cancer Control Act and related government promotional efforts, the number of PCTs in Japan has been increasing rapidly.

Although the functioning of PCTs is superior with respect to facilitating collaboration among several specialists and the introduction of palliative care services at early stages, the number of PCTs in Japan remains low due to the aforementioned strict requirements. A survey of all Japanese university hospitals (*N *= 123) conducted in 2005 found that 33% contained uncertified PCTs, and 11% contained certified PCTs[15]. It is regrettable that more than half of the university hospitals in Japan did not offer PCTs even though these institutions are expected to act as pioneers and set the standards for all kinds of hospitals. However, no certified palliative care program is offered by community or certified home-based palliative care specialists in Japan, as it is in Western countries.

In addition, confusion about the role of PCTs also relates to several practical issues. Many Japanese hospitals are adopting a system in which a primary physician adopts total control of the life and care of patients from the beginning of an illness until discharge or death. Japanese physicians are not yet familiar with the methods underpinning continual collaboration with PCTs because this approach originated abroad, and some physicians harbour misconceptions about PCTs (e.g., that these units will not take any responsibility for patients). To our knowledge, few studies have examined the barriers to PCTs and the negative attitudes toward continual collaboration with PCTs among physicians. Therefore, we investigated the factors that determine physicians' attitudes about the role of PCTs. As Dunlop stated, "it should be a very rare exception that the primary team hands over the responsibility for patients" and "PCT members must act as role models rather than take over care;"[19] indeed, the key to palliative care is continual collaboration between physicians and PCTs. In addition, we believe that PCTs should adopt a consultation model to increase palliative care referrals. It is necessary to conduct an empirical study to determine how to promote PCTs from the perspective of the consultation model. Therefore, this study surveyed physicians' attitudes toward collaboration with PCTs and described the factors relevant to these attitudes.

## Methods

This was a cross-sectional anonymous questionnaire-based survey. The initial sample consisted of 339 physicians, including interns, who were employed full-time at a large hospital with 30 medical departments and 925 beds located in a metropolitan area in Japan. The hospital is one of the national centres administrated by the Japanese government and provides general acute care to those in its geographic area. The hospital's department of palliative care includes full-time physicians and has provided PCT services since 2003. We excluded physicians who were not involved in primary care, such as radiologists and pathologists. The department heads distributed the questionnaires to the physicians in their departments. The survey was conducted from 2 February to 9 February 2007.

### Study participants

**Figure 1:**
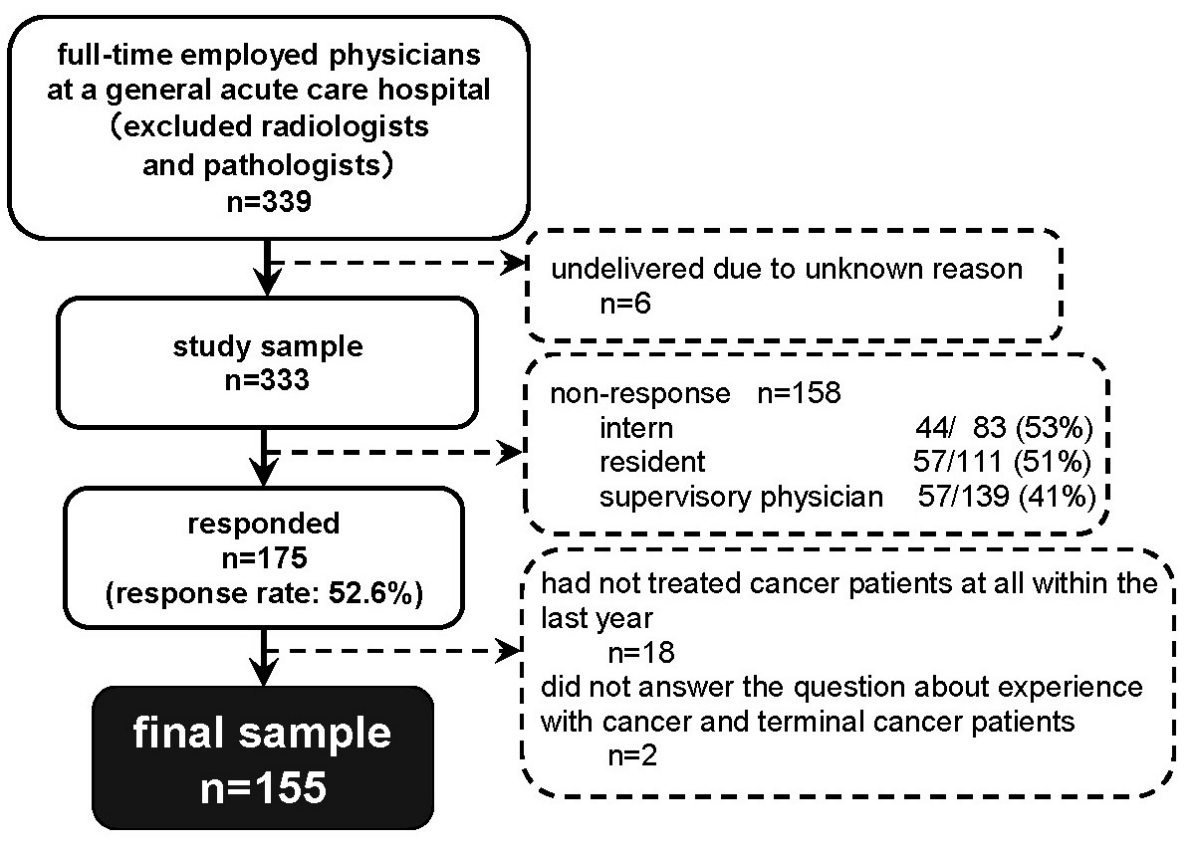
**Study population and sampling procedure**.

Three hundred and thirty-nine full-time physicians were identified as potential participants but, for reasons unknown, six questionnaires were not delivered. Of the 333 questionnaires sent to the physicians, 175 were returned (response rate: 53%). Of those returned, 18 physicians had not treated any cancer patients within the last year. Because the hospital provided palliative care services primarily to cancer patients (i.e., rather than to patients with other diagnoses), and because most physicians in Japan believe that PCTs are appropriate for the treatment of cancer patients, we excluded these 18 physicians as well as two physicians who did not answer the question about experience with cancer and terminal cancer patients. A total of 155 responses were analyzed (Figure 1).

The study sample included 104 males (67%), 51 females (33%), and the mean age of the sample was 32.0 years (range: 25-65 years). The sample included 38 interns (25%), 47 residents (30%), and 70 supervisory physicians (45%), and the average number of years in practice was 6.0 (range: 1-40). Of the total number of physicians who received questionnaires (*n *= 333), 83 were interns (25%), 111 were residents (33%), and 139 were supervisory physicians (42%); we found no significant differences in these proportions in the final sample (25%, 30%, and 45%, respectively, *p *= 0.744 according to the *chi*-square test). Therefore, the study sample can be considered representative of all physicians in this hospital with respect to their positions.

### Main outcome and related factors

We used the physicians' attitudes toward continual collaboration with the PCTs as the outcome variable, which was measured by responses to the following items: (1) "I want to treat and care for my patients without collaboration," (2) "I want to treat and care for my patients until the end, but I also want to collaborate with the PCT," (3) "I want to treat and care for my patients until the end, but I want the PCT to be in charge," and (4) "I want to leave the responsibility to palliative care physicians." Item (2) indicates that the PCT is considered to play a supportive role in the treatment team, and (3) indicates that the PCT occupies the main position in the team; both items imply engaging in continual collaboration with the PCT. Item (4) implies a negative attitude toward continual collaboration with the PCT in that it reflects the opinion that only palliative care specialists are able to provide palliative care.

The independent variables included physician characteristics, experience, knowledge, and education. We also collected data on age, sex, position as a physician, duration of experience as a physician, interest in palliative care ("Are you are interested in palliative care?"), and concerns about death, which consisted of the summary of the scores on four items from the Death Attitude Inventory ("You often think about what death is," "You often think about your death," "You often think about the death of a person who is close to you," and "You often talk about death with your family and friends")[20]. The last two items were assessed on a scale from 1 to 7 in which 1 corresponded to "never" and 7 corresponded to "always."

We collected data on experiences related to cancer patients and palliative care in terms of requests for PCT consultation ("Yes" or "No"); training in providing medical care at home ("Yes" or "No"); actually providing medical care at home ("Yes" or "No"); and the level of communication with patients and family members about place of care ("You discuss the place of care with the patient/patient's family"), place of death ("You discuss the place of death with the patient/patient's family"), symptoms of dying ("You talk with the patient/patient's family about the symptoms and physical changes that occur during the final stage of death"), and resuscitation ('You talk with the patient/patient's family about do-not-resuscitate (DNR) orders")[21]. The level of communication was assessed on a 7-point scale similar to that used to measure interest in palliative care and concerns about death.

Knowledge and education about the WHO analgesic ladder were also evaluated, as in a previous study[19]. Participants were asked about their knowledge of the WHO analgesic ladder ("Have you ever heard about the WHO analgesic ladder?"), and respondents expressing familiarity with this document were then asked to select all answers that applied regarding where they acquired this knowledge ("Where did you learn about it?) in a closed-ended question ("at medical school, during postgraduate education, at a conference or workshop, from a paper or technical book, through PCT consultation, or other").

### Statistical analyses

We used Fisher's exact test for dichotomous variables for univariate analysis related to outcome. The Wilcoxon rank-sum test was used for continuous variables. The level of significance for differences was set at *p *= < 0.05, two-sided.

To determine the factors contributing to physicians' attitudes toward continual collaboration with PCTs, we used multiple logistic regression to estimate the adjusted odds ratios (OR) and 95% confidence intervals (95% CI). We used Spearman's correlation coefficient to exclude the effect of collinearity among the independent variables emerging from univariate analyses as significant in terms of the outcome measure. Several factors obtained after excluding the effect of multicollinearity were initially included in the model, and a stepwise analysis was then performed.

All analyses were performed using SAS software (Windows Version, Release 8.02; SAS Institute, Cary, NC, USA). The study protocol was reviewed and approved by the institutional review board and ethics committees of the University of Tsukuba.

## Results

### Continual collaboration with the PCT

In response to the question, "What do you think about collaboration with the PCT?", one participant (0.6%) answered "I want to treat and care for my patients without collaboration" (1), 82 (52.9%) responded "I want to treat and care for my patients until the end, but I also want to collaborate with the PCT" (2), 42 (27.1%) endorsed "I want to treat and care for my patients until the end, but I want the PCT to be in charge" (3), 20 (12.9%) said "I want to leave the responsibility to the PCT" (4), and 10 (6.5%) said that they did not know or did not respond.

We divided the physicians into two groups according to their responses: those expressing positive attitudes toward ongoing collaboration with the PCT [i.e., responses (2) or (3)] and those who wanted to leave responsibility to the PCT [i.e., response (4)]. The basic characteristics of the two groups are presented in Table 1, and their backgrounds related to cancer patients and palliative care are summarized in Table 2 in terms of experience, knowledge, and education.

**Table 1:** Basic characteristics of study participants according to attitudes toward continual collaboration with PCTs (median with range, or number with %)

		"Continual collaboration"		
			
		Positive *n *= 124^a^	Negative *n *= 20^a^	*P*-value
Age (in years)		32 (25-65)	38 (29-62)	0.01
Sex	male	80 (65%)	18 (90%)	0.04
	female	44 (36%)	2 (10%)	
Position	intern^b^	37 (30%)	0 (0%)	0.01^e^
	resident^c^	32 (26%)	7 (35%)	
	supervisory physician^d^	54 (44%)	13 (65%)	
Physician experience (in years)		5 (1--40)	11 (3--37)	0.01
Interest in palliative care (scores)^f^		6 (1--7)	4 (2--7)	<0.01
Concerns about death (scores)^g^		16 (4--28)	16 (4--28)	0.46

^a ^The totals do not match because of missing values.

^b ^Approximately 1-2 years after receiving their medical licenses.

^c ^Approximately 3-5 years after receiving their medical licenses.

^d ^Approximately 6 years or more after receiving their medical licenses.

^e ^Analysis of interns and residents versus supervisory physicians.

^f ^Answers ranged from "none" (1 point) to "definite" (7 points) with respect to interest in palliative care.

^g ^Concerns about death was selected from the Death Attitude Inventory and each item was rated on a scale from "applicable" (1 point) to "inapplicable" (7 points), and scores on concerns about death reflect total points for the four items.

**Table 2:** Experience, knowledge, and education of study participants according to attitudes toward continual collaboration with PCTs

		Continual collaboration		
			
		Positive ***n *= 124**^a^	Negative ***n *= 20**^a^	*P*-value
**Experience:**				
Request PCT consultation		99 (80%)	13 (65%)	0.15
Training in medical care at home		54 (44%)	4 (20%)	0.05
Medical care at home		49 (40%)	8 (40%)	1.00
Communication (mean and median with ranges)^b^:				
place of care	with patients	5.4, 6 (1--7)	5.1, 5 (2--7)	0.37
	with family	6.1, 7 (1--7)	5.3, 6 (2--7)	0.03
place of death	with patients	4.4, 4 (1--7)	3.2, 4 (1--7)	0.01
	with family	5.7, 6 (1--7)	4.4, 4 (1--7)	0.01
symptoms of dying	with patients	4.3, 4 (1--7)	3.3, 4 (1--7)	0.04
	with family	5.7, 6 (1--7)	4.9, 5 (1--7)	0.04
DNR	with patients	4.3, 4 (1--7)	4.1, 4 (1--7)	0.75
	with family	6.4, 6 (1--7)	6.2, 7 (1--7)	0.31
**Knowledge:**				
About WHO analgesic ladder		99 (80%)	6 (39%)	<0.01
**Education: **(among subjects with knowledge of the WHO analgesic ladder)				
Where knowledge about WHO analgesic ladder was acquired^c^:				
medical school		43 (43%)	3 (50%)	0.12
postgraduate education		34 (34%)	5 (83%)	1.00
conference or workshop		8 (8%)	1 (17%)	1.00
paper or technical book		23 (23%)	2 (33%)	0.53
through PCT consultation		25 (25%)	2 (23%)	0.37

^a ^The totals do not match because of missing values.

^b^Answers ranged from "inapplicable" (1 point) to "applicable" (7 points).

^c^Multiple answers were allowed and each item was analyzed in terms of "yes" vs. "no" responses.

### Physician characteristics

The group with positive attitudes toward continual collaboration with PCTs ("positive group," *n *= 124) was younger (*p *= 0.01) and included more females (*p *= 0.04) than did the other group ("negative group," *n *= 20) (Table 1). The positive group included significantly more interns and residents (56%), and the physicians in this group were significantly less experienced (mean of 9.5 and median 5 years) compared with those in the negative group (35%, mean of 14.4 and median 11 years, respectively) (*p *= 0.01). The positive group expressed significantly greater interest in palliative care (mean: 5.7, median: 6) than did the negative group (mean: 4.5, median: 4) (*p *< 0.01), but no significant difference emerged with respect to concerns about death (*p *= 0.46) (Table 1).

### Experience, knowledge, and education

Members of the positive group were significantly more likely to have received training in performing medical care at home (44%) than were members of the negative group (20%) (*p *= 0.05), and the positive group communicated well with patients' families about the site of care (*p *= 0.03), the place of death (*p *= 0.01), and the symptoms of dying (*p *= 0.04) (Table 2). Similar positive levels of communication were observed with respect to place of death (*p *= 0.01) and symptoms of dying (*p *= 0.04). However, the groups did not differ significantly with regard to other experiences, such as requesting PCT consultation and providing medical care at home.

The groups showed significant differences in knowledge about the WHO analgesic ladder; significantly more physicians in the positive group (80%) than in the negative group (39%) were aware of this resource (*p *< 0.01). We found no significant difference between the positive and negative groups in terms of where such knowledge was acquired (Table 2).

### Factors contributing to attitudes toward continual collaboration with PCTs

We analyzed 12 variables and present those with significant differences in Tables 1 and 2. Length of experience as a physician was strongly correlated with age and position. For this reason, we included physician experience in the logistic regression analysis as a representative variable related to attitudes toward continual collaboration with PCTs. Similarly, discussion with family members about place of death was chosen to indicate the level of communication with patients and family members. Therefore, six variables (physician sex, experience, interest in palliative care, training in providing medical care at home, discussion about place of death with family members, and knowledge about the WHO analgesic ladder) were initially included in the model. As a result of a stepwise process, four factors were included in the final model: physician sex, experience, knowledge about the WHO analgesic ladder, and interest in palliative care.

We found that physicians who favoured continual collaboration were more likely to be aware of the WHO ladder (OR = 6.75, 95% CI = 1.98-25.79) than were those who did not favour collaboration (Table 3). Similarly, physicians who favoured continual collaboration were more likely to be interested in palliative care (OR = 1.68, 95% CI = 1.15-2.50).

**Table 3:** Multivariate odds ratios (OR) and 95% confidence intervals (CI) for the association between positive attitudes toward continual collaboration with PCTs and independent variables

Independent variables	OR	(95% CI)
Sex (male)	2.58	(0.57--18.6)
Physician experience (years)	0.99	(0.93--1.06)
Knowledge about WHO analgesic ladder (Yes)	6.75	(1.98--25.8)
Interest in palliative care (scores)^a^	1.68	(1.15--2.50)

^a ^Answers ranged from "none" (1 point) to "definite" (7 points) with respect to interest in palliative care.

## Discussion

This study found that physicians who were aware of the WHO ladder were 6.8 times more likely to want to treat and care for their patients in collaboration with PCTs than were physicians without such awareness even when adjusted for the level of their interest in palliative care and other possible confounding factors, such as physician's sex and length of experience. These results indicate that physicians' positive attitudes toward continual collaboration with PCTs were strongly associated with basic knowledge about palliative care. Few studies have demonstrated a relationship between a preference for continual collaboration with PCTs and factors that significantly contribute to this preference.

The WHO analgesic ladder is just one element in the basic knowledge involved in palliative care, but it includes important ideas that can improve patients' QOL. Di Maio *et al*.[22] conducted a survey of 1,021 patients with advanced non-small-cell lung cancer and found that patients in their sample frequently experienced pain that significantly affected their QOL. A 10-year prospective study conducted by Zech *et al*.[23] found that the WHO ladder remained a consistently effective approach to pain and other clinical symptoms and suggested that the QOL of patients can be improved when their physicians are knowledgeable about basic palliative care. Moreover, Morita *et al*.[6] observed that PCTs were introduced at more appropriate times and evaluated as more useful for symptom control than were PCUs. Therefore, knowledge of the WHO analgesic ladder is indispensable for the provision of adequate palliative care by physicians, and basic knowledge seems to change physicians' attitudes toward continual collaboration with PCTs.

Although this study included only one institution, our results can be generalized to many hospitals and physicians because our subjects were general physicians working in an acute-care hospital. Unlike end-of-life patients in hospice and home care, most cancer patients in acute-care hospitals receive aggressive cancer treatment. The palliative care of patients in a hospice or those receiving home care usually involves physicians continuously collaborating with PCTs. In recent years, despite efforts by physicians to provide early analgesic treatment to patients receiving aggressive cancer treatment, such patients and their families have tended to regard palliative care negatively, and these feelings have resulted in the relatively late introduction of palliative interventions,[3,4] thereby leading to a vicious cycle. The tendency of patients to refuse palliative care is caused by the lack of knowledge about analgesia and the misconception that this approach will result in the deterioration of patients' QOL. Our results suggest the possibility of improving patients' QOL when physicians have appropriate knowledge about palliative care and engage in continual collaboration with the PCT in their acute-care hospital. Such physicians can educate patients and their families about the importance of palliative care and can use the specialized skills of PCTs to address each patient's symptoms at the appropriate time.

In addition, the number of PCTs in Japan has been increasing rapidly owing to the Cancer Control Act and related government promotional efforts, and as a result, the problems associated with the late introduction of palliative interventions are now better known in acute-care hospitals[4,6]. According to our results, basic knowledge about palliative care is sufficient for developing a positive attitude toward continual collaboration with PCTs, which will facilitate the introduction of analgesic treatment to patients at appropriate times. However, no system for acquiring this basic knowledge during undergraduate or postgraduate education was identified. Thus, we would like to recommend that basic knowledge regarding palliative care should be reviewed with physicians working in acute-care hospitals equipped with PCTs.

Within the context of a medical institution, physicians' attitudes toward PCTs are very important. Even when nurses are interested in clinical palliative care, the attitudes of physicians wield great influence on the therapeutic strategy pursued because decision-making authority for treatment and care belongs to physicians. Thus, if more physicians become knowledgeable about palliative care and come to favour continual collaboration with PCTs, the number of possible therapeutic strategies will increase. As a result, medical staff will be able to provide effective treatment and care for their patients by focusing on the symptoms present at each stage of illness. A wide range of treatment approaches is likely to contribute to the QOL of patients receiving aggressive treatment in acute-care hospitals.

This study has several limitations. First, the nonresponse rates for interns (53%), residents (52%), and supervisory physicians (41%) were relatively high (Figure 1). Although this difference did not reach statistical significance, physicians with less status (interns and residents) had lower response rates. Given that younger physicians work longer hours,[24] it is possible that these low response rates are attributable to lower-status physicians' being too busy with clinical work to answer our questionnaire. This would suggest that the study sample was biased in favour of less busy physicians. Moreover, participants interested in palliative care and those who were able to collaborate with PCTs on a more continuous basis than were average physicians might have been more willing to answer all the questions. These sampling biases need to be considered when the results of this study are applied in other settings.

Second, this study did not consider differences in departments. Physicians in particular departments, such as those involving gastrointestinal conditions, would be expected to be familiar with the analgesic ladder because they encounter cancer patients more frequently and treat them for longer periods than do physicians in other departments. As a result, they might have more experience with PCTs or be more aware of the importance of continual collaboration with PCTs. Therefore, differences in departments might have acted as a confounding variable.

## Conclusions

Despite these limitations, this study offers important benefits because it surveyed general physicians about their attitudes to collaboration with PCTs. The development of leaders in this field is necessary for encouraging physicians to specialize in palliative care and to acquire basic knowledge about this domain, including about the WHO analgesic ladder.

## Competing interests

The authors declare that they have no competing interests.

## Authors' contributions

NT was the chief investigator for the project and provided final approval for the study design of the project. MO conducted the survey, performed data analysis, interpreted the data, and wrote the draft version of this article. MK was involved in the development of the project and revised the article with respect to important intellectual content. MN conceived the study, helped in its implementation, analyzed the focus groups, and contributed to the manuscript. EA made the final decision to develop the project and arranged for the recruitment of participants. All authors read and approved the final manuscript.

## Pre-publication history

The pre-publication history for this paper can be accessed here:

http://www.biomedcentral.com/1472-684X/9/13/prepub
